# Extracellular cyclophilin A possesses chemotaxic activity in cattle

**DOI:** 10.1186/s13567-015-0212-1

**Published:** 2015-07-11

**Authors:** Satoru Takanashi, Tomonori Nochi, Miku Abe, Nanami Itaya, Megumi Urakawa, Katsuyoshi Sato, Tao Zhuang, Saori Umemura, Tomohito Hayashi, Yoshio Kiku, Haruki Kitazawa, Michael T. Rose, Kouichi Watanabe, Hisashi Aso

**Affiliations:** Laboratory of Mucosal Immunology, Graduate School of Agricultural Science, Tohoku University, Miyagi, 981-8555 Japan; International Education and Research Center for Food and Agricultural Immunology, Graduate School of Agricultural Science, Tohoku University, Miyagi, 981-8555 Japan; National Agriculture and Food Research Organization, National Institute of Animal Health, Hokkaido, 062-0045 Japan; Food and Feed Immunology Group, Graduate School of Agricultural Science, Tohoku University, Miyagi, 981-8555 Japan; Institute of Biological, Environmental and Rural Sciences, Aberystwyth University, Cardiganshire, SY23 3DA UK

## Abstract

Cyclophilin A (CyPA) was originally discovered in bovine thymocytes as a cytosolic binding protein of the immunosuppressive drug cyclosporine A. Recent studies have revealed that in mice and humans, CyPA is secreted from cells in injured or infected tissues and plays a role in recruiting inflammatory cells in those tissues. Here we found that in cattle abundant level of extracellular CyPA was observed in tissues with inflammation. To aid in investigating the role of extracellular CyPA in cattle, we generated recombinant bovine CyPA (rbCyPA) and tested its biological activity as an inflammatory mediator. When bovine peripheral blood cells were treated with rbCyPA in vitro, we observed that rbCyPA reacts with the membranous surface of granulocytes, monocytes and lymphocytes. Chemotaxis analysis showed that the granulocytes migrate toward rbCyPA and the migration is inhibited by pre-treatment with an anti-bovine CyPA antibody. These results indicate that, as for mice and humans, extracellular CyPA possesses chemotactic activity to recruit inflammatory cells (e.g., granulocytes) in cattle, and could thus be a potential therapeutic target for the treatment of inflammation.

## Introduction

Inflammation occurs when tissues are injured by exposure to pathogens (e.g., bacteria, viruses) or foreign substances (e.g., toxins, chemicals) [1]. Chemokines, which are small cytokines sharing a basic structure composed of three anti-parallel β-strands and an overlying α-helix, play a key role in regulating leucocyte trafficking into inflammatory tissues [2]. So far, nearly 50 kinds of chemokine belonging to either the CXC, CC, C or CX3C families have been identified; all have important roles, not only in inflammation but also homeostatic responses [3]. However, recent studies have shown that, in addition to chemokines, other factors without the structural characteristics peculiar to the chemokines, are also involved in leucocyte trafficking as they also possess potent chemotactic activity [4].

Cyclophilins, which consist of 16 members in humans, are a family of peptidyl prolyl *cis-trans* isomerases [5,6]. Cyclophilin A (CyPA), which is the most abundant member of the family, has multiple biological roles in protein folding, trafficking, T cell activation and cell signaling [5]. CyPA also acts as the intracellular receptor for the immunosuppressive drug cyclosporin A [7]. CyPA had been believed to be present only in the intracellular space, but recent studies have shown that it is secreted from cells in response to hypoxia, infection and oxidative stress [8]. An example is that the reactive oxygen species induces CyPA secretion from vascular smooth muscle cells (VSMCs), resulting in the formation of abdominal aortic aneurysms (AAA), in which abnormal proliferation of VSMCs, unusual expression of endothelial cell adhesion molecule, and aberrant infiltration of inflammatory cells in the aortic wall are observed [9,10].

CD147 (also known as extracellular matrix metalloproteinase inducer; EMMPRIN) has been known to act as the main signaling receptor for extracellular CyPA [11]. Leucocyte migration toward CyPA is essentially regulated by CD147 [11–14]. In mice CD147 expression is found on lymphocytes, monocytes and granulocytes present in peripheral blood [12], such that these cells could recognize CyPA and migrate into injured tissues where CyPA is secreted. Treatment with anti-CD147 monoclonal antibody inhibits CyPA-mediated inflammatory cell recruitment [12]. Therefore, interference of CyPA-CD147 interaction would be a novel potential therapeutic to reduce infiltration of inflammatory cells into injured tissues.

Inflammation caused by infection with bacteria or viruses in cattle, such as during mastitis and pneumonia, is a serious problem in the dairy cattle industry because such diseases are directly linked to a significant financial loss [15,16]. Numerous strategies have been developed for the prevention and cure of these diseases [17,18], but their incidence across the industrialized world is still high, and therefore more research is still required in order to reduce the mortality and morbidity caused. Moreover, more precise mechanisms by which inflammatory cells migrate into the injured tissues from peripheral blood in cattle must be addressed.

Here we show that abundant levels of extracellular CyPA is found in the mammary gland during mastitis. Using recombinant bovine CyPA (rbCyPA), we demonstrate that extracellular bovine CyPA is involved in recruiting inflammatory cells (e.g., granulocytes). These results indicate that extracellular CyPA could possess chemotaxic activity to induce inflammation in cattle.

## Materials and methods

### Animal studies

Mammary tissue (*n* = 6) and peripheral blood cells (*n* = 3) were obtained from 9 Holstein dairy cows. All animal studies were performed in accordance with protocols approved by Tohoku University Institutional Animal Care and Use Committee.

### Immunohistochemistry

Mammary gland tissues collected from cattle with (*n* = 3) and without (*n* = 3) mastitis were fixed in 4% (w/v) of paraformaldehyde (PFA) or periodate lysine paraformaldehyde (PLP) overnight at 4 °C, and embedded in paraffin. Tissue sections (3 μm) were first subjected to antigen retrieval using a REAL™ target retrieval solution (DAKO). After blocking with 3% (v/v) of normal goat serum (Vector), the sections were stained with 1 μg/mL of rabbit anti-mouse/rat/human CyPA antibody (Abcam, Catalog No: ab41684, hereafter called the commercial anti-CyPA antibody), which is predicted to react to bovine CyPA according to the manufacturer’s data sheet, or 1 μg/mL of control rabbit immunoglobulin (Dako). The sections were then stained with Histofine® Simple Stain MAX PO (R) (Nichirei Biosciences) and the signal was developed with 3,3′-diaminobenzidine tetrahydrochloride (DAB). Finally, counterstaining with hematoxylin was performed.

### Recombinant bovine CyPA (rbCyPA)

The gene coding bovine CyPA was synthesized by PCR with PrimeStar® HS DNA polymerase (Takara) and specific primers (sense, 5′-TAGCTCAAGCTTTGATGGTCAACCCCACCGTGTTCTTC-3′; antisense, 5′-CGCTCGCTCGAGTTAGATTTGTCCACAGTCAGCAAT-3′; HindIII and XhoI restriction enzyme sites are shown by underlining). PCR fragment and the expression vector pPAL7 (Bio-Rad) were digested with HindIII and XhoI, and were ligated with DNA ligation kit ver. 2.1 (Takara). After DNA sequencing, the plasmid was used to transform BL21 (DE3) (Bio-Rad), and the cells were treated with 0.1 mM of isopropyl-β-D-thiogalactopyranoside (IPTG) for 4 h at 37 °C to induce Profinity eXact-tagged bovine CyPA expression. The cells were then suspended in PBS and sonicated. After centrifugation, the supernatant was loaded onto a column packed with Profinity eXact™ Purification Resin (Bio-Rad) that has enzymatic activity to cleave between the Profinity eXact tag and rbCyPA. Tag-free rbCyPA obtained by affinity chromatography was finally loaded onto a Sephacryl S-100 column (GE Healthcare) to prepare highly purified rbCyPA. SDS-PAGE and western-blotting with the commercial anti-CyPA antibody (Abcam) or control rabbit immunoglobulin (Dako) were performed to confirm the expression and purification of rbCyPA. A portion of the rbCyPA was conjugated with fluorescein isothiocyanate (FITC), Isomer I on Celite (Sigma), which has the thiocyanate group that crosslinks with amino group on rbCyPA. To determine whether the reactivity of the commercial anti-CyPA antibody (Abcam) was neutralized by pre-incubation with rbCyPA, 1 mg/mL of rbCyPA was treated with the 10 μg/mL of the commercial anti-CyPA antibody for 1 h at room temperature (RT), and the mammary gland tissue sections were treated with the antigen-antibody complex after dilution with PBS (1:10, final antibody concentration was 1 μg/mL, consistent with the regular staining protocol for immunohistochemistry).

### Binding analysis

Bovine peripheral blood was obtained from jugular vein and heparinized. Erythrocytes were lysed using 0.83% (w/v) of ammonium chloride. The cells were suspended in PBS (1.0 × 10^6^ cells /100 μL) and incubated with 10 μg/mL of either rbCyPA or bovine serum albumin (BSA) (Sigma), both of which were pre-conjugated with FITC (Sigma), for 10, 60, 120, 240 and 360 min at 37 °C. After incubation, flow cytometry data were collected using a BD Accuri™ C6 flow cytometer (BD Bioscience), and analyzed 1 × 10^4^ cells using BD Accuri™ C6 software (BD Bioscience) and FCS Express 5 (De Novo Software). Mouse anti-bovine granulocyte antibody (MM20A, mouse IgG1, 20 μg/mL), mouse anti-bovine CD14 antibody (DG-CAN36A, mouse IgG1, 20 μg/mL), mouse anti-bovine CD3 antibody (MM1A, mouse IgG1, 20 μg/mL) and isotype control mouse IgG1 (COLIS69A, 20 μg/mL), all of which were purchased from Washington State University Monoclonal Antibody Center, were used to develop a gating strategy for the binding analysis with FITC-conjugated rbCyPA and FITC-conjugated BSA. Via-Probe™ (BD Bioscience) was used to exclude dead cells.

### Rabbit anti-bovine CyPA polyclonal antibody

A rabbit was immunized with rbCyPA 6 times every week at Eurofins Genomics K.K. to obtain large amount of anti-bovine CyPA polyclonal antibody, enough for an inhibition study. This was because the commercial anti-CyPA antibody (Abcam) was not available in sufficient amounts for testing our hypothesis that a CyPA-targeted therapeutic could be an effective strategy for the treatment of inflammation. The first three immunizations were all performed subcutaneously with 200 μg of rbCyPA plus Freund’s complete adjuvant, and the last three immunizations were performed intravenously with 25, 50 and 50 μg of rbCyPA, respectively. Serum samples were collected before the first immunization and after the final booster. After IgG purification with Protein G (GE Healthcare), these serum samples were for use as the control antibody and anti-bCyPA antibody, respectively. IgG titers of anti-bCyPA antibody and control antibody against rbCyPA were measured by ELISA. Briefly, 96-well ELISA plates (Nunc) were coated with rbCyPA (2 μg/mL, 100 μL) overnight at 4 °C. After blocking with 1% BSA in TBST [50 mM Tris–HCl (pH 8.0), 0.05% Tween-20] for 1 h at RT, diluted IgG antibodies were incubated for 2 h at RT. After washing, the plates were treated with HRP-conjugated donkey anti-rabbit IgG (Jackson Immunoresearch) diluted 1:20 000 for 1 h at RT and the signals were finally developed with a TMB microwell peroxidase substrate system (KPL).

### Chemotaxis analysis

Granulocytes were used for a chemotaxis analysis with rbCyPA. In brief, peripheral blood mononuclear cells in cattle (*n* = 2) were removed by centrifugation with Lympholyte-H (Cedarlane) and erythrocytes were then lysed with 0.83% (w/v) of ammonium chloride to obtain the granulocytes. To confirm their purity, flow cytometry analysis with mouse anti-bovine granulocyte antibody (MM20A, Washington State University Monoclonal Antibody Center) was performed. For the chemotaxis analysis, a cell migration assay using Transwell® inserts with 5.0 μm pore polycarbonate membrane (Corning) were performed, as described previously [19]. Briefly, granulocytes suspended in STEMPRO® (Gibco, 1.0 × 10^6^ cells/cm^2^/100 μL) were cultured in the upper chambers of the inserts and several different concentrations (0, 30, 50, 100, 300 or 500 ng/mL, 600 μL) of rbCyPA were added into the lower chambers (24 well plates). In some experiments, rbCyPA (100 ng/mL) was pre-incubated with 100 μg/mL of either anti-bCyPA antibody or the control antibody for 60 min at RT. After incubation for 90 min at 37 °C in a 5% CO_2_ incubator, the membranes of the transwell® inserts were stained with Giemsa reagent (Nacalai tesque). In an each experimental condition, 15 or 16 images (76 800 μm^2^ / image) were obtained from 3 or 4 membranes using a microscope AX70 (Olympus) and the numbers of granulocytes migrating to the lower chamber of the insert were counted manually.

### Statistical analysis

All statistical analyses (alpha level: 0.01) were performed in Prism version 6 (GraphPad). Two-way repeated measure ANOVA and Tukey’s multiple comparison test were performed for Figure 3b, One-way factorial ANOVA and Tukey’s multiple comparison tests were performed for Figures 4c and 5c.

## Results

### Secretion of CyPA in mammary gland of cattle with mastitis

**Figure 1:**
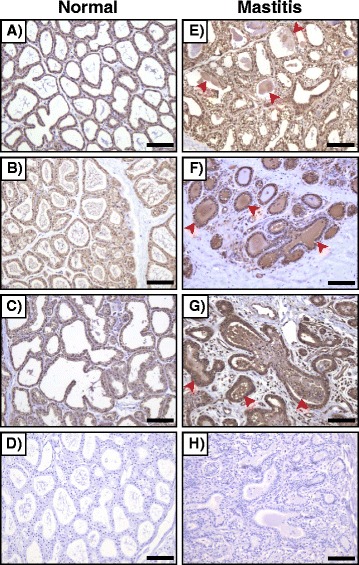
**CyPA is secreted in cattle during inflammation.** Immunohistochemical analysis shows that extracellular CyPA is abundantly found in alveoli and ducts of bovine mammary gland with mastitis (shown by *arrowheads*). **A**-**C** and **E**-**G** (both of which were stained with anti-CyPA antibody) are images showing individual healthy cattle and individual cattle with mastitis, respectively. **D** (healthy) and **H** (mastitis) are images stained with control antibody. Scale bar = 100 μm.

As CyPA in mice and humans has been known to be secreted from cells during inflammation, such as abdominal aortic aneurysms (AAA), our initial study was designed to investigate whether extracellular CyPA was also found in cattle during inflammation. When mammary gland tissues sampled from cattle with or without mastitis were analyzed by immunohistochemistry with the commercial anti-CyPA antibody, we found that, regardless of the presence of mastitis, CyPA was present in the cytoplasm of mammary epithelial cells (Figure 1). Importantly, however, abundant levels of extracellular CyPA were only detected in the alveoli and ducts in mammary tissues from mastitic cattle (Figure 1). We also found that inflammatory cells expressing CyPA were highly recruited in stromal tissue of mammary gland with mastitis. No signals were detected in sections stained with the control rabbit immunoglobulin (Figure 1). These results indicate that, as in mice and humans, CyPA is secreted from cells in cattle during inflammation, such as mastitis.

### Expression and purification of recombinant bovine CyPA (rbCyPA)

**Figure 2:**
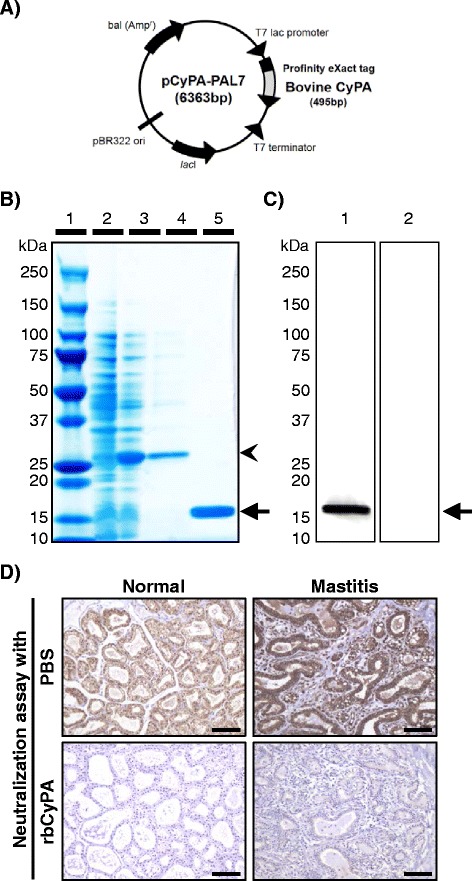
**Recombinant bovine CyPA (rbCyPA) is generated in an** ***E.coli*** **expression system.** **A** pbCyPA-PAL7 (6363 bp) was constructed to express rbCyPA in *E.coli* (BL21). **B** SDS-PAGE analysis showed that Profinity eXact-tagged rbCyPA was expressed when BL21 cells were treated with IPTG and obtained as a soluble protein. Tag-free rbCyPA was collected after affinity and gel chromatography. Lane 1: molecular weight marker, 2: Cell lysate of BL21 cells cultured without IPTG, 3: Cell lysate of BL21 cultured with IPTG, 4: Soluble proteins obtained by sonication of cell lysate of BL21 cultured with IPTG, 5: rbCyPA collected after affinity and gel chromatography. **C** Western-blot analysis was conducted using the commercial anti-CyPA antibody (1) or the control rabbit immunoglobulin (2) to confirm that the band shown on lane 5 of Figure 2B was bovine CyPA. **D** Neutralization with rbCyPA completely inhibited the immunoreactivity of anti-CyPA antibody used for immunohistochemistry. Scale bars = 100 μm.

In order to address the role of extracellular CyPA in cattle, rbCyPA was produced and its biological activity was determined. An expression plasmid (pbCyPA-PAL7, 6363 bp), in which the bovine CyPA gene was inserted in the downstream of Profinity eXact tag sequence, was constructed (Figure 2A). *E. coli* (BL21) was transformed with pbCyPA-PAL7 and treated with IPTG to induce the expression of Profinity eXact-tagged bovine CyPA. SDS-PAGE analysis showed that one major band of approximately 26 kDa was detected only when the BL21 cells were cultured with IPTG (Figure 2B, lanes 2 and 3). Importantly, the band was still observed when the BL21 cells were sonicated and the supernatant was loaded on the SDS-PAGE gel (Figure 2B, lane 4). These results indicated that the vast majority of the Profinity eXact-tagged bovine CyPA expressed in BL21 cells was a soluble protein. After affinity and gel chromatography with the supernatant, we observed a single band of approximately 16 kDa (Figure 2B, lane 5). Subsequent western-blot analyses showed that the band was recognized when the commercial anti-CyPA antibody, not control rabbit immunoglobulin, was used (Figure 2C). We also confirmed by a neutralizing study that the reactivity of the commercial anti-CyPA antibody used for immunohistochemistry (shown in Figure 1) was completely blocked by pretreatment with the generated rbCyPA (Figure 2D). Given that Profinity eXact™ Purification Resin used for affinity chromatography has the enzymatic activity to cleave between Profinity eXact tag and rbCyPA [20], tag-free rbCyPA obtained was used for the next in vitro analyses in order to address the role of extracellular CyPA in cattle.

### Binding analysis with rbCyPA

**Figure 3:**
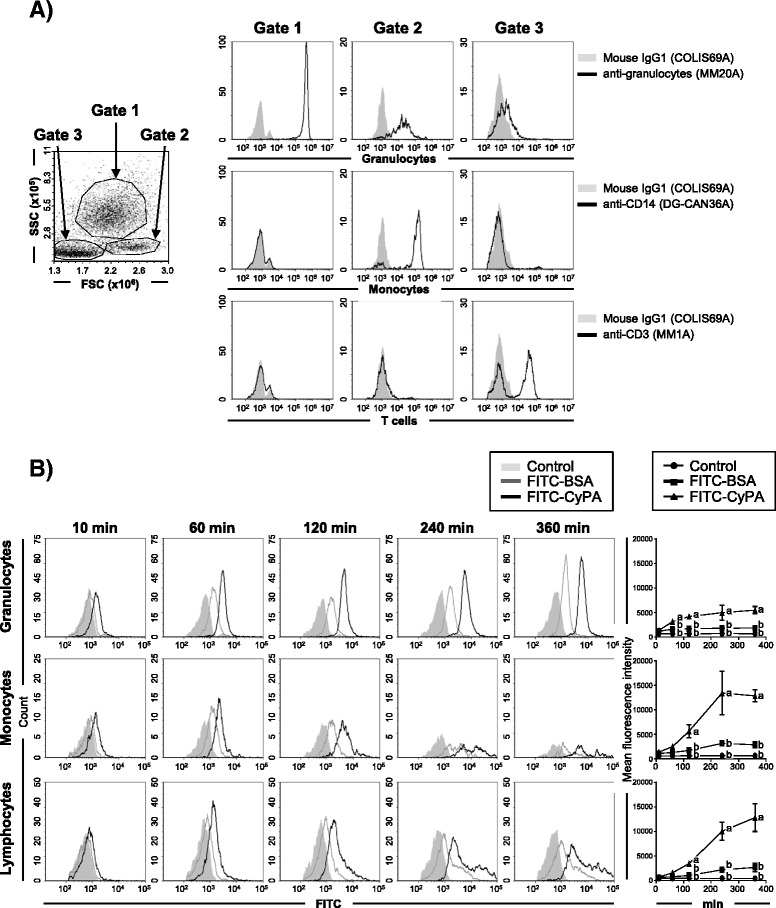
**rbCyPA binds to granulocytes, monocytes and lymphocytes present in bovine peripheral blood. A** A gating strategy for flow cytometry analysis was established by staining with anti-bovine granulocytes, CD14 or CD3. Three major populations (gate 1, 2 and 3) were found on the FSC and SSC profiles of bovine peripheral blood cells (1 left panel). Cells present in gate 1, 2 and 3 were granulocytes, monocytes and lymphocytes, respectively, because these were granulocyte^+^CD14^−^CD3^−^ cells, granulocyte^low^CD14^+^CD3^−^ cells and granulocyte^−^CD14^+^CD3^+ or –^ cells (9 right panels), respectively. **B** rbCyPA reacted to granulocytes, monocytes and lymphocytes in a time-dependent manner. Longitudinal change is shown in the line graph on the right. Three separate experiments were performed and the data represent means ± SEM. Values with different letters are statistically different (*p* < 0.01).

In order to investigate the reaction of rbCyPA with immune cells in cattle, we performed an in vitro binding analysis with rbCyPA. Immune cells collected from bovine peripheral blood were first analyzed by flow cytometry using three antibodies: anti-bovine granulocyte antibody (MM20A), which reacts highly to granulocytes and slightly with monocytes; anti-bovine CD14 antibody (DG-CAN36A), which specifically binds to monocytes; and anti-bovine CD3 antibody (MM1A), which reacts with T cells. This was to develop a gating strategy for the in vitro binding analysis. We observed that granulocyte^+^CD14^−^CD3^−^ cells, granulocyte^low^CD14^+^CD3^−^ cells and granulocyte^−^CD14^+^CD3^+ or –^ cells, which correspond to granulocytes, monocytes and lymphocytes, respectively, were observed in three major populations; these were named gate 1, gate 2 and gate 3 on a FSC and SSC profile (Figure 3A). These results indicated that the gating strategy based on the FSC and SSC profile could be used for distinguishing the three major cell types (i.e., granulocytes, monocytes and lymphocytes) without any additional time restriction for staining using anti-bovine granulocyte, anti-bovine CD14 and anti-bovine CD3 antibodies after in vitro study. To address whether extracellular CyPA reacts to immune cells in cattle, we next prepared FITC-conjugated rbCyPA (FITC-CyPA) and used it for an in vitro binding analysis. We found that, depending on the reaction time, the reactivity of rbCyPA to all three cell types was increased gradually (Figure 3B). Importantly, the reactivity to immune cells was higher than that of FITC-conjugated BSA (FITC-BSA) used as a control (Figure 3B). These results indicated that the receptor for extracellular CyPA could be present in all of the immune cells analyzed.

### Chemotaxis analysis with rbCyPA

**Figure 4:**
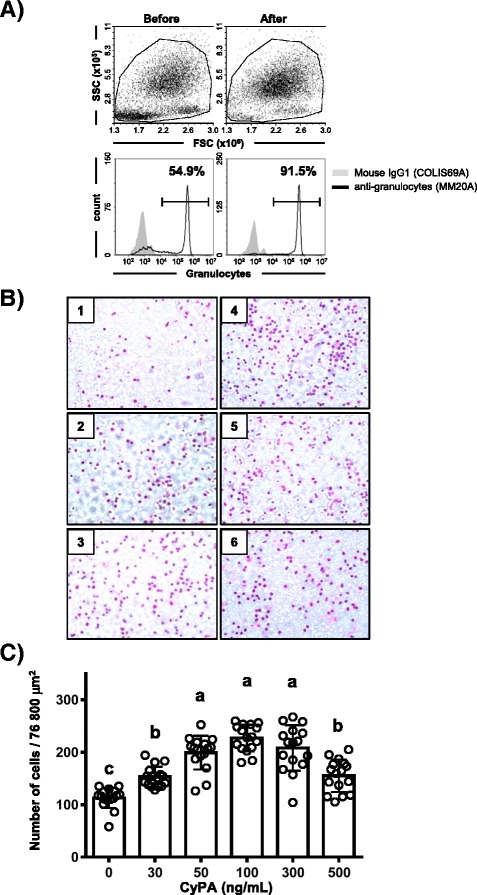
**rbCyPA possesses chemotactic activity.** **A** Granulocytes were purified by centrifugation with Lympholyte-H and the purification was confirmed by staining with anti-granulocyte antibody. **B** Chemotactic analysis with several different concentration of rbCyPA was performed. Granulocytes migrating toward rbCyPA are shown. 1: 0 ng/mL, 2: 30 ng/mL, 3: 50 ng/mL, 4: 100 ng/mL, 5: 300 ng/mL, 6: 500 ng/mL of rbCyPA. **C** Chemotactic activity was evaluated by counting of granulocytes that migrated toward rbCyPA. Three separate experiments were performed and one representative result is shown. Values with different letters are statistically different (*p* < 0.01).

In order to analyze the role of extracellular CyPA in cattle, we performed a chemotaxis analysis with rbCyPA. Among the three major white blood cell populations (granulocytes, monocytes and lymphocytes) present in peripheral blood (Figure 3A), we focused on investigating granulocytes because neutrophils, which are the major population among granulocytes, initially migrate into injured tissues to induce inflammatory responses [21]. When we enriched granulocytes from peripheral blood cells by density-gradient centrifugation with Lympholyte-H, the ratio of granulocytes recognized by anti-bovine granulocyte antibody exceeded 90% (Figure 4A). We next cultured the granulocytes in the upper chambers of Transwell® inserts and stimulated the cells with several different concentrations of rbCyPA added to the lower chambers. We found that up to a concentration of 50 ng/mL of rbCyPA, the granulocytes migrated toward rbCyPA in a dose-dependent manner (Figures 4B and C). Interestingly, when concentrations of greater than 50 ng/mL were tested, there was no further increase in migration up to a concentration of 300 ng/mL; above a concentration of 500 ng/mL, the proportion was decreased (Figures 4B and C). These results indicate that 50–300 ng/mL of rbCyPA is the optimal concentration to induce maximal rbCyPA-mediated chemotactic activity in granulocytes.

### Inhibition of rbCyPA-mediated chemotaxis by anti-bCyPA antibody

**Figure 5:**
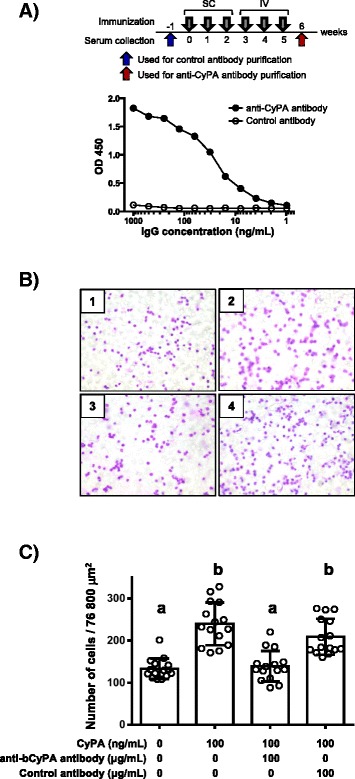
**rbCyPA-mediated chemotactic activity was inhibited by the use of anti-bovine CyPA antibody. A** Rabbit anti-bovine CyPA polyclonal antibodies were generated by immunization with rbCyPA. Serum was collected both before the first immunization (*arrow*) and after the final booster (*red arrow*) to obtain the control and anti-bovine CyPA antibodies, respectively. Bovine CyPA-specific antibody titers were measured by ELISA. SC: subcutaneous injection, IV: intravenous injection. **B** and **C** Chemotactic analysis was performed after pre-treatment with 100 ng/mL of rbCyPA with 100 μg/mL of either the anti-bovine CyPA antibody or control antibody. rbCyPA-mediated chemotactic activity was inhibited by pre-treatment with anti-bovine CyPA antibody (not control antibody). **B** 1: no rbCyPA + no antibody, 2: 100 ng/mL of rbCyPA + no antibody, 3: 100 ng/mL of rbCyPA + 100 μg/mL of anti-bovine CyPA antibody, 4: 100 ng/mL of rbCyPA + 100 μg/mL of control antibody. **C** Three separate experiments were performed and one representative result is shown. Values with different letters are statistically different (*p* < 0.01).

We next designed a neutralizing study with an anti-bovine CyPA antibody in order to consider the future possibility of CyPA-targeted anti-inflammatory therapeutics. A rabbit was immunized with rbCyPA 6 times and the antiserum was collected 1 week after the final immunization (red arrow in Figure 5A). As a control, serum was also collected 1 week before the first immunization (blue arrow in Figure 5A). A high titer of bCyPA-specific IgG antibody was observed when IgG purified from antiserum was analyzed by ELISA with rbCyPA-coated plates (Figure 5A). In contrast, no detectable level of anti-bCyPA antibody was found when IgG purified from control serum was examined (Figure 5A). Thus, in our next study, we used IgG purified from both antiserum and control serum as anti-bCyPA antibody and control antibody, respectively. Given that 50–300 ng/mL of rbCyPA is the optimal concentration to induce a maximal effect of rbCyPA-mediated chemotactic activity in granulocytes (Figures 4B and C), we decided to neutralize 100 ng/mL of rbCyPA with 100 μg/mL of either anti-bCyPA antibody or control antibody. Consistent with our previous study, as shown in Figures 4B and C, 100 ng/mL of rbCyPA showed an efficient chemotactic activity to granulocytes. However, pretreatment with the anti-bCyPA antibody inhibited the rbCyPA-mediated chemotactic activity completely (Figures 5B and C). In contrast, granulocyte migration toward rbCyPA was observed as normal when rbCyPA pre-treated with control antibody was used (Figures 5B and C). Taken together, the neutralization of extracellular CyPA function could be a potent immunotherapy for the prevention and cure of inflammation across a range of diseases in cattle.

## Discussion

CyPA is an evolutionarily conserved protein that is present in the cytoplasm of all prokaryotes and eukaryotes [6]. The amino acid sequence homology among human, mouse and bovine CyPA is high (98.8%: bovine vs human; 96.3%: human vs mouse; 95.1%: bovine vs mouse), and the predicted molecular mass of human, mouse and bovine CyPA are almost identical (human: 18.0 kDa, mouse: 18.0 kDa and bovine: 17.9 kDa). Recent studies have shown that CyPA is secreted from cells in response to inflammatory stimuli and oxidative stress, and that the role of extracellular CyPA differs from that of intracellular CyPA in mice and humans [5]. We also found that abundant level of extracellular CyPA is present in tissues with inflammation in cattle (Figure 1), but investigations to gain an insight into the role of extracellular bovine CyPA have not yet been conducted. Therefore, in this study, we first generated recombinant bovine CyPA (rbCyPA) using an *E. coli* expression vector pPAL7 (Figure 2A). The use of pPAL7 allows us to generate tag-free protein without any extra non-related amino acid residues, as Profinity eXact™ Purification Resin used for the affinity chromatography has enzymatic activity to cleave just before the first methionine [20], which is the start codon of rbCyPA. SDS-PAGE and western-blot analyses showed that highly purified rbCyPA without any contaminated proteins was obtained by affinity and gel chromatography (Figures 2B and C). As a note, approximately 20 mg of purified rbCyPA was collected from 20 liters of BL21 cell culture. Given this sufficient quality and quantity of purified rbCyPA, we decided to use it for our subsequent in vitro studies.

CD147, is a type I transmembrane protein and has been known to act as a receptor for extracellular CyPA in mice and humans [11–14]. CD147 is ubiquitously expressed on many types of murine immune cells (e.g., lymphocytes, monocytes and granulocytes), but no studies have been conducted yet to demonstrate the expression of CD147 on bovine immune cells due to the lack of a commercially available anti-bovine CD147 antibody. Nevertheless, our alternative approach, using rbCyPA conjugated with FITC (FITC-CyPA), indicated that lymphocytes, monocytes and granulocytes present in bovine peripheral blood have a receptor for extracellular CyPA because FITC-CyPA reacted to all immune cells analyzed, in a time-dependent manner when incubated in vitro (Figure 3B). We have not yet obtained direct evidence to show that the receptor for extracellular CyPA is CD147 in cattle, but this experiment suggests that the binding molecule of extracellular CyPA is expressed by bovine lymphocytes, monocytes and granulocytes.

Neutrophils initially migrate into injured (or damaged) tissues to initiate inflammatory responses [21]. In a mouse model of acute lung inflammation, the level of secretion of CyPA is increased dramatically when lipopolysaccharide is administered intra-nasally [12]. The increased level of CyPA leads to CD147-mediated neutrophil recruitment into the lung tissues, such that treatment with anti-CD147 antibody in this mouse model inhibits the CyPA-CD147 interaction, resulting in reduced tissue neutrophilia with a concurrent decrease in tissue pathology [12]. Therefore, the first priority of our study was to investigate the role of extracellular CyPA for neutrophils in cattle. Among the cell types: neutrophils, eosinophils and basophils (all categorized as granulocytes), neutrophils are the most abundant cell population [22]. Therefore, we used whole enriched granulocytes, the purity of which was more than 90% (Figure 4A). We found a dose-dependent and bell-shaped curved reaction of rbCyPA to bovine granulocytes (Figures 4B and C). Moreover, the optimal concentration that induced the maximum chemotactic activity was 50–300 ng/mL (Figures 4B and C), which is consistent with a previous study showing the chemotactic activity of extracellular CyPA in mice [12]. These results indicate that the role of extracellular CyPA is highly conserved across species.

Antibody administration in patients to prevent the function of pathogenic factors (e.g., proinflammatory cytokines) or to attack the abnormal cells (e.g., cancer cells) is widely known as an effective immunotherapy [23]. For example, anti-human tumor necrosis factor (TNF) alpha-specific monoclonal antibody or anti-human CD20-specific monoclonal antibody has been extensively used as an innovative medicine for patients with rheumatoid arthritis or leukemia [24,25]. Inhibition of extracellular CyPA function using CyPA-specific neutralizing antibodies has been also considered to be a potent immunotherapy, because extracellular CyPA recruits inflammatory cells to accelerate the abnormal immune responses [8]. Moreover, in our study, we demonstrated that the function of extracellular CyPA is also common to the bovine. Therefore, to confirm our hypothesis that extracellular CyPA could be a therapeutic target to prevent the recruitment of inflammatory cells in cattle, we generated rabbit anti-bovine CyPA polyclonal antibody. When 100 ng/mL of rbCyPA, which is predicted to induce a maximum chemotactic activity, was pre-treated with 100 μg/mL of rabbit anti-bovine CyPA polyclonal antibody, the chemotactic activity was completely removed (Figures 5B and C). We have not yet estimated the financial aspects of a CyPA-targeted therapy using an antibody in cattle. However, given the result obtained in this proof-of-principal study showing the inhibitory effect for the inflammatory cell influx through extracellular CyPA, not only antibody therapies but also other chemotherapeutics that inhibit the role of extracellular CyPA could be considered as a practical approach to prevent extracellular CyPA-mediated inflammation in cattle.

We demonstrated here that extracellular CyPA possesses chemotactic activity to recruit inflammatory cells in cattle, and treatment with anti-bovine CyPA antibody is capable of inhibiting the extracellular CyPA-mediated inflammatory cell trafficking. Taken together, extracellular CyPA could be a potential therapeutic target for the treatment and cure of inflammation in cattle.

## Abbreviations

AAA: Abdominal aortic aneurysms
CyPA: Cycrophilin A
DAB: 3,3′-diaminobenzidine tetrahydrochloride
ELISA: Enzyme*-*lnked immunosorbent assay
FITC: Fluorescein isothiocyanate
FSC: Forward Scatter
HRP: Horseradish peroxidase
IPTG: Isopropyl-β-D-thiogalactopyranoside
PFP: Paraformaldehyde
PLP: Periodate lysine paraformaldehyde
SSC: Side Scatter

## References

[1] Buckley CD, Gilroy DW, Serhan CN, Stockinger B, Tak PP (2013). The resolution of inflammation. Nat Rev Immunol.

[2] Homey B, Muller A, Zlotnik A (2002). Chemokines: agents for the immunotherapy of cancer?. Nat Rev Immunol.

[3] Bendall L (2005). Chemokines and their receptors in disease. Histol Histopathol.

[4] Bukrinsky MI (2002). Cyclophilins: unexpected messengers in intercellular communications. Trends Immunol.

[5] Nigro P, Pompilio G, Capogrossi MC (2013). Cyclophilin A: a key player for human disease. Cell Death Dis.

[6] Wang P, Heitman J (2005). The cyclophilins. Genome Biol.

[7] Handschumacher RE, Harding MW, Rice J, Drugge RJ, Speicher DW (1984). Cyclophilin: a specific cytosolic binding protein for cyclosporin A. Science.

[8] Yurchenko V, Constant S, Eisenmesser E, Bukrinsky M (2010). Cyclophilin-CD147 interactions: a new target for anti-inflammatory therapeutics. Clin Exp Immunol.

[9] Satoh K, Nigro P, Matoba T, O’Dell MR, Cui Z, Shi X, Mohan A, Yan C, Abe J, Illig KA, Berk BC (2009). Cyclophilin A enhances vascular oxidative stress and the development of angiotensin II-induced aortic aneurysms. Nat Med.

[10] Satoh K, Nigro P, Berk BC (2010). Oxidative stress and vascular smooth muscle cell growth: a mechanistic linkage by cyclophilin A. Antioxid Redox Signal.

[11] Yurchenko V, Zybarth G, O’Connor M, Dai WW, Franchin G, Hao T, Guo H, Hung HC, Toole B, Gallay P, Sherry B, Bukrinsky M (2002). Active site residues of cyclophilin A are crucial for its signaling activity via CD147. J Biol Chem.

[12] Arora K, Gwinn WM, Bower MA, Watson A, Okwumabua I, MacDonald HR, Bukrinsky MI, Constant SL (2005). Extracellular cyclophilins contribute to the regulation of inflammatory responses. J Immunol.

[13] Gwinn WM, Damsker JM, Falahati R, Okwumabua I, Kelly-Welch A, Keegan AD, Vanpouille C, Lee JJ, Dent LA, Leitenberg D, Bukrinsky MI, Constant SL (2006). Novel approach to inhibit asthma-mediated lung inflammation using anti-CD147 intervention. J Immunol.

[14] Damsker JM, Bukrinsky MI, Constant SL (2007). Preferential chemotaxis of activated human CD4^+^ T cells by extracellular cyclophilin A. J Leukoc Biol.

[15] Czuprynski CJ (2009). Host response to bovine respiratory pathogens. Anim Health Res Rev.

[16] Schukken YH, Gunther J, Fitzpatrick J, Fontaine MC, Goetze L, Holst O, Leigh J, Petzl W, Schuberth HJ, Sipka A, Smith DG, Quesnell R, Watts J, Yancey R, Zerbe H, Gurjar A, Zadoks RN, Seyfert HM (2011). Host-response patterns of intramammary infections in dairy cows. Vet Immunol Immunopathol.

[17] Erskine RJ (2012). Vaccination strategies for mastitis. Vet Clin North Am Food Anim Pract.

[18] Bennett N, Ellis J, Bonville C, Rosenberg H, Domachowske J (2007). Immunization strategies for the prevention of pneumovirus infections. Expert Rev Vaccines.

[19] Liu L, Li C, Xiang J, Dong W, Cao Z (2013). Over-expression and potential role of cyclophilin A in human periodontitis. J Periodontal Res.

[20] Imaizumi K, Nishikawa S, Tarui H, Akuta T (2013). High-level expression and efficient one-step chromatographic purification of a soluble human leukemia inhibitory factor (LIF) in Escherichia coli. Protein Expr Purif.

[21] Kolaczkowska E, Kubes P (2013). Neutrophil recruitment and function in health and inflammation. Nat Rev Immunol.

[22] Wang J, Zhou X, Pan B, Yang L, Yin X, Xu B, Zhao D (2013). Investigation of the effect of Mycobacterium bovis infection on bovine neutrophils functions. Tuberculosis (Edinb).

[23] Yamane-Ohnuki N, Satoh M (2009). Production of therapeutic antibodies with controlled fucosylation. MAbs.

[24] Feldmann M (2002). Development of anti-TNF therapy for rheumatoid arthritis. Nat Rev Immunol.

[25] Jain P, O’Brien S (2013). Anti-CD20 monoclonal antibodies in chronic lymphocytic leukemia. Expert Opin Biol Ther.

